# Data retrieval from archival renal biopsies using nonlinear microscopy

**DOI:** 10.1371/journal.pone.0299506

**Published:** 2024-03-15

**Authors:** Lucas C. Cahill, Tadayuki Yoshitake, Milan Rosen, Timothy D. Weber, James G. Fujimoto, Seymour Rosen

**Affiliations:** 1 Harvard-MIT Division of Health Sciences and Technology, Harvard Medical School and Massachusetts Institute of Technology, Cambridge, MA, United States of America; 2 Department of Electrical Engineering and Computer Science and Research Laboratory of Electronics, Massachusetts Institute of Technology, Cambridge, MA, United States of America; 3 Department of Pathology, Beth Israel Deaconess Medical Center, Harvard Medical School, Boston, MA, United States of America; Colorado State University, UNITED STATES

## Abstract

Thorough examination of renal biopsies may improve understanding of renal disease. Imaging of renal biopsies with fluorescence nonlinear microscopy (NLM) and optical clearing enables three-dimensional (3D) visualization of pathology without microtome sectioning. Archival renal paraffin blocks from 12 patients were deparaffinized and stained with Hoechst and Eosin for fluorescent nuclear and cytoplasmic/stromal contrast, then optically cleared using benzyl alcohol benzyl benzoate (BABB). NLM images of entire biopsy fragments (thickness range 88–660 μm) were acquired using NLM with fluorescent signals mapped to an H&E color scale. Cysts, glomeruli, exudative lesions, and Kimmelstiel-Wilson nodules were segmented in 3D and their volumes, diameters, and percent composition could be obtained. The glomerular count on 3D NLM volumes was high indicating that archival blocks could be a vast tissue resource to enable larger-scale retrospective studies. Rapid optical clearing and NLM imaging enables more thorough biopsy examination and is a promising technique for analysis of archival paraffin blocks.

## Introduction

Thorough examination of the renal biopsy enabling classification and mapping of disease will be a major part of the effort to improve clinical therapies and understand renal disease [1]. This could be especially important in diseases that have a focal injury pattern (i.e. diseases that affect the minority of glomeruli on a biopsy when analyzed by histology). Current standard histologic sampling techniques analyze only a small fraction of the renal biopsy. This means that a substantial amount of biopsy information is missed and large stores of archival paraffin blocks are available for analysis.

Differentiating between minimal change disease (MCD) and focal segmental glomerulosclerosis (FSGS) is important for predicting therapeutic response and disease prognosis [2]. Both diseases present with proteinuria, diffuse podocyte effacement, and minimal immune deposits under immunofluorescence and electron microscopy studies [2]. MCD has unremarkable changes in paraffin histology, while FSGS is characterized by sclerotic lesions in a small population of glomeruli, at least in initial phases [3]. The more glomeruli that are evaluated in paraffin histology, the higher possibility that a glomeruli affected by FSGS will be found [4]. A 17% increase in the number of glomeruli found to be involved with FSGS was found when evaluating biopsies via serial sections compared to the standard-of-care sectioning [5]. Further, accurately determining the extent of involved glomeruli has important implications for pathogenesis and potential therapeutic response [5].

Other focal diseases including anti-neutrophil cytoplasmic antibody (ANCA) glomerulonephritis (GN) often present as focal and segmental necrotizing and crescentic glomerulonephritis [6]. It has been reported that 73% of glomeruli in focal ANCA-associated GN are normal while only 14% of glomeruli have segmental crescentic lesions, and 6.5% have globally sclerotic lesions [7]. Having high proportions of normal tissue in renal biopsies makes it difficult to consistently quantify disease severity or prognosis based on two dimensional paraffin slides (interobserver and intraobserver variability is high) [8].

Systemic lupus erythematosus (SLE) nephritis can be focal and the interpretation and classification of the disease has poor reproducibility between pathologists [9]. In particular, differentiating SLE class II, a purely mesangial process, from SLE class III, which affects glomerular capillaries, can be difficult when limited tissue is evaluated [10]. Furthermore, classifications are based off of the extent of glomerular involvement which can be misrepresented when subsampling the tissue [11]. These difficulties and inconsistencies have led to a call for revisiting and reclassifying SLE nephritis [11].

Diabetic nephropathy is another disease where focal lesions are an important part of disease classification. In diabetic nephropathy, the presence of Kimmelstiel-Wilson nodules defines Class III disease while lower classes are absent of these lesions.

Complete, three-dimensional (3D) analysis of tissue specimens has been shown to enhance information on disease and tissue pathology [12,13], however, this often requires laborious serial sectioning [14,15]. Studies have performed 3D evaluation of whole animal organs embedded in paraffin by combining nonlinear microscopy (NLM) with serial microtome sectioning to overcome the depth penetration limitations of NLM imaging [16]. Combining tissue clearing with microscopy techniques such as NLM enables 3D analysis of tissue volumes without physical sectioning. These techniques have been demonstrated to improve examination of renal tissue from animals [17–20] and human cadavers [21].

Olson, et al. [21] described a method of optically clearing simulated renal biopsies from fixed human autopsies with benzyl alcohol benzyl benzoate (BABB) and staining with 4’,6-diamidino-2-phenylindole and Eosin. Their clearing and staining methods are fast and enable visualization of tissue similar to H&E histology. In this study, similar clearing and staining methods are used to extend the findings of these previous studies by demonstrating the tissue processing and imaging methods on true archival renal biopsies from patients with common renal diseases such as diabetic nephropathy, IgA nephropathy, focal segmental glomerular sclerosis, and acute tubular necrosis. Renal paraffin blocks are deparaffinized and stained with Hoechst and Eosin, then optically cleared using benzyl alcohol benzyl benzoate (BABB). 3D NLM imaging is performed and displayed using an H&E color scale. Preliminary demonstrations quantifying glomeruli and tissue pathology are presented as future parameters for larger-scale clinical studies. Furthermore, compatibility with standard histology stains such as Periodic acid–Schiff (PAS), Jones methenamine silver, and Masson’s trichrome stains is verified.

## Materials and methods

### Tissue preparation

Archival renal biopsy paraffin blocks from 12 patients were collected. All research was performed according to Institutional Review Board protocols.

The paraffin blocks were melted and processed in a reverse cycle on a vacuum infiltrating processor (10 minutes at 65°C, 9 xylene washes, 5 ethanol washes, 1 water wash) to remove paraffin from the tissue. The tissue clearing method was based on a method described by Dent, *et al*. [22] and Olson, *et al*. [21] Solutions of increasing concentration of methanol (70% for 1 hour, 95% and 100% for 30 minutes each) with 40 μg/mL Hoechst 33342 (Life Technologies, Eugene, OR) and 1 μL/mL eosin Y (5% by weight in water, Sigma-Aldrich, St. Louis, MO) in each solution were used to dehydrate biopsies and label nuclear and cytoplasmic/stromal components. Combining tissue staining with dehydration reduces tissue preparation time. The biopsies were rinsed in 100% methanol for 15 minutes to remove excess dye. Then the biopsies were optically cleared in a 2:1 solution of benzyl alcohol (ACS reagent, >99%, Sigma-Aldrich, St. Louis, MO,): benzyl benzoate (ReagentPlus®, >99%, Sigma-Aldrich, St. Louis, MO) (BABB) for 4 to 6 hours. Staining and clearing was performed at 40°C on a shaker table to improve penetration.

### NLM imaging and image processing

After staining and tissue clearing, the biopsies were placed on a glass coverslip (No. 1.5, VWR, Radnor, PA) and biopsy foam (30.2 x 24.5 x 2 mm (M476), Beloeil, Quebec, Canada) was used to gently maintain the biopsy’s position against the glass surface.

A previously described nonlinear microscope [23] was used to evaluate the renal biopsies. A Ti:Sapphire femtosecond laser (Mira, Coherent, Santa Clara, CA) operated at 780 nm was scanned with resonant and galvanometer scanners through a 20X, 1.0 NA water immersion objective (XLUMPFL20XW, Olympus, Tokyo). A 525 nm high-pass dichroic beam splitter (T525lpxr, Chroma Technology Corporation, Bellows Falls, VT) split the Hoechst and Eosin fluorescent emission light through two separate emission filters (FF02-460/80-25 and FF01-590/104-25, Semrock, Rochester, NY) onto two photomultiplier tubes (H7422P-40, Hamamatsu, Hamamatsu City, Japan). 1024 x 1024 pixel frames were acquired over a 0.49 x 0.49 mm area. Entire biopsy cross sections were acquired by automatically translating the biopsy with a precision linear motor stage (MLS203, Thorlabs, Newton, NJ) and imaging overlapping frames at a single depth from the biopsy surface. Biopsy cross sections were then acquired every 4 μm in depth by translating the specimen in the specimen in the depth with a piezo stage (MZS500-E, Thorlabs), generating a 3D NLM dataset.

The tissue preparation (alcohol dehydration, staining, and clearing) required 6–8 hours. The NLM acquisition time varied depending on the size of the biopsy. The duration of imaging ranged from 10 minutes (450-frame dataset) to 5.5 hours (18,000-frame dataset). The microscope used was not optimized for speed and we expect that future version will provide large reductions in acquisition times.

The NLM images were mapped into an H&E color scale using an algorithm called Virtual Transillumination Microscopy [24]. The frames in each biopsy cross section were stitched together using Image Composite Editor (Microsoft, Redmond, WA) so that they could be viewed analogously to a whole slide image from a digital slide scanner. 3D volumes were reconstructed using Amira (ThermoFisher, Waltham, MA).

After NLM imaging, biopsies were placed in 100% methanol for 1 hour to remove the BABB. The specimens were then re-processed in a vacuum infiltrating processor and re-embedded in paraffin. H&E, PAS, Jones methenamine silver, and Masson’s trichrome paraffin embedded sections were made.

### Analysis, segmentation, and counting

A renal pathologist with over 20 years of experience (SR) read the NLM images using OpenSeadragon, a web-based viewer that enables variable magnification review. Similarities and differences between the NLM images, paraffin histology, and original archival biopsy pathology report were investigated. Cysts, glomeruli, exudative lesions, and Kimmelstiel-Wilson nodules were manually segmented in sequential 2D planes of the 3D NLM volumes using Amira. Glomeruli were counted on the 3D NLM volumes. No automated segmentation or analysis algorithms were used in this initial study.

### Tissue evaluated in the original pathology report vs NLM imaging

To make the original diagnoses, pathologists typically evaluated 10–15 slides with 2–4 sections (2–3 μm thickness) per slide. The renal biopsies, after fixation, had a diameter of ~1 mm. To make the histology slides, serial sections (directly adjacent tissue) were not always used on adjacent slides. Instead sections were sampled through the block and much of the tissue was wasted. All tissue that was left in the block after microtome sectioning for paraffin histology was evaluated by NLM.

## Results

### Nonlinear microscopy images of renal biopsies: Specific conditions

Results from 12 biopsies obtained from archival paraffin blocks are presented. Biopsies were examined from patients with diabetic nephropathy, IgA nephropathy, focal and segmental glomerulosclerosis (FSGS) with features of collapsing glomerulopathy, acute tubular necrosis, thrombotic microangiopathy, global sclerosis, interstitial fibrosis, vascular disease, and advanced chronic kidney disease. NLM enables 3D evaluation of biopsy specimens, generating images that closely resemble paraffin H&E sections.

#### Diabetic nephropathy

Biopsies from four patients diagnosed with diabetic nephropathy were analyzed. NLM images corresponded closely to the paraffin H&E slides and enabled 3D visualization and analysis of many of the characteristic features of diabetic nephropathy.

**Fig 1:**
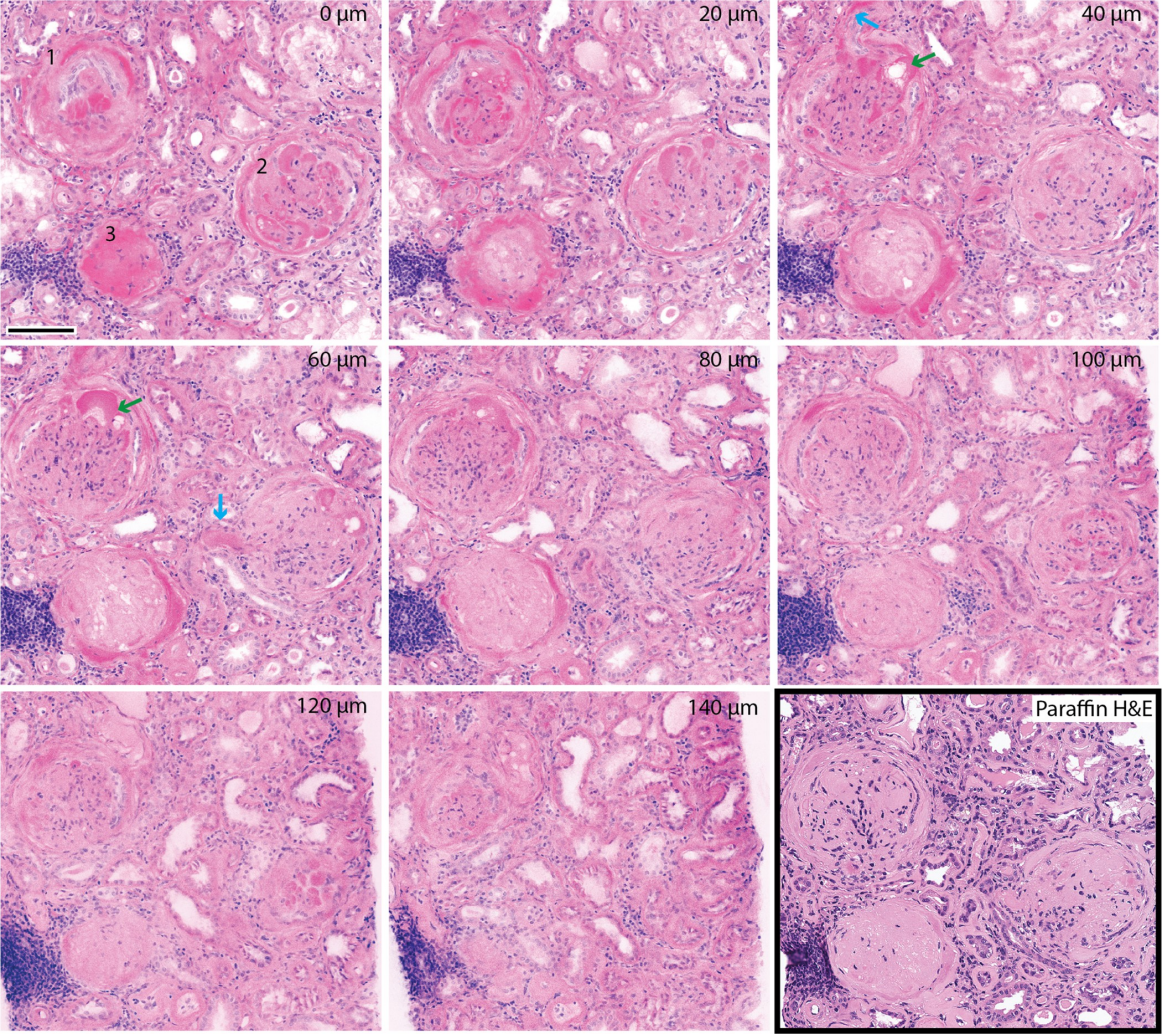
Three-dimensional NLM analysis of a biopsy from a patient with advanced diabetic nephropathy. NLM images were acquired every 4 μm in depth. Images spaced 20 μm apart in depth show advanced glomerular changes in 3 different glomeruli (labelled 1, 2, and 3). Capillary lumens and Bowman spaces are occluded with reduction of the glomerular tuft to a collapsed acellular mass of basement membranes and matrix. Exudative lesions are abundant, have a vacuolated (lipid) component (green arrow), and can be seen as both part of the glomerular tuft and Bowman’s capsule, the latter forming an encompassing ring. The hyalinized arterioles are evident in images at the 40 μm and 60 μm level (blue arrow). The depth below the biopsy surface is labelled in top right of each image. Scale bar = 100 μm.

Fig 1 shows a region of interest from a biopsy of a patient with advanced diabetic nephropathy in NLM images selected at different levels. Three glomeruli are present showing varying degrees of disease involvement. Glomerulus 1 is characterized by prominent endocapillary exudative lesions that are also present in Bowman’s capsular area. The urinary space is largely occluded and associated with some degree of proliferation. Endocapillary cellularity is irregularly reduced. At deeper levels, Bowman’s space is almost entirely lost and the glomerular tuft exudative lesions continue to be prominent and have a vacuolated (lipid) component (green arrow). In Glomerulus 2, the glomerular tuft in the first level (0 μm) has prominent exudative lesions with some degree of maintained endocapillary cellularity. At deeper levels, the urinary space becomes totally obliterated. Hyalinized arterioles are seen at the 40 μm and 60 μm level of Glomerulus 1 and 2, respectively (blue arrow). Glomerulus 3 shows even more advanced changes. The exudative lesions are now encircling the tuft, which is basically acellular matrix plus basement membranes. A corresponding paraffin H&E slide of the same region is shown (bottom right).

**Fig 2:**
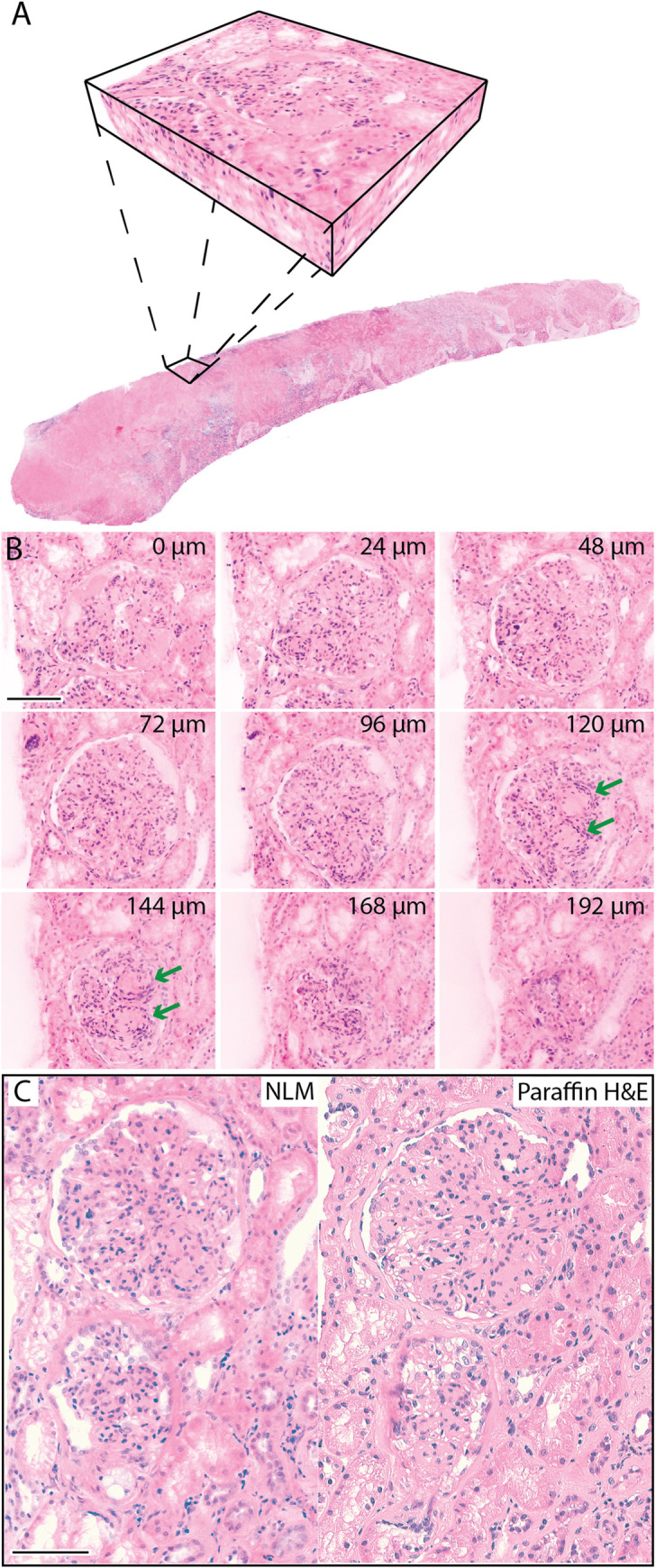
Three-dimensional NLM analysis of a biopsy from a patient diagnosed with diabetic nephropathy and IgA nephropathy. (**A**) NLM images were acquired every 4 μm in depth through the biopsy and reconstructed into a 3D volume. (**B**) NLM images selected at different levels of a glomerulus. The depth below the tissue surface is indicated in microns (top right). **(C)** An NLM image and corresponding paraffin H&E slide. Hypercellularity and mesangial expansion are evident in the NLM images and paraffin H&E slide, but the Kimmelstiel-Wilson nodules are only clearly defined at the NLM levels 120 μm and 144 μm (B, green arrow). Scale bar = 100 μm.

Fig 2A shows an example of a 3D NLM volume of a biopsy from a patient with diabetic and IgA nephropathy. Fig 2B shows a glomerular tuft displayed in 24 μm intervals. Glomerular mesangial expansion is seen by hypercellularity and matrix expansion, which occludes capillary lumens. Kimmelstiel-Wilson nodules are seen only at levels 120 μm and 144 μm. Thus, in a situation of mesangial expansion and hypercellularity (IgA), morphologically characteristic lesions are more clearly defined by 3D imaging. An NLM image and a corresponding paraffin H&E slide of this biopsy are shown in Fig 2C. Mesangial expansion and hypercellularity are seen in both images.

#### Focal and segmental glomerulosclerosis

**Fig 3:**
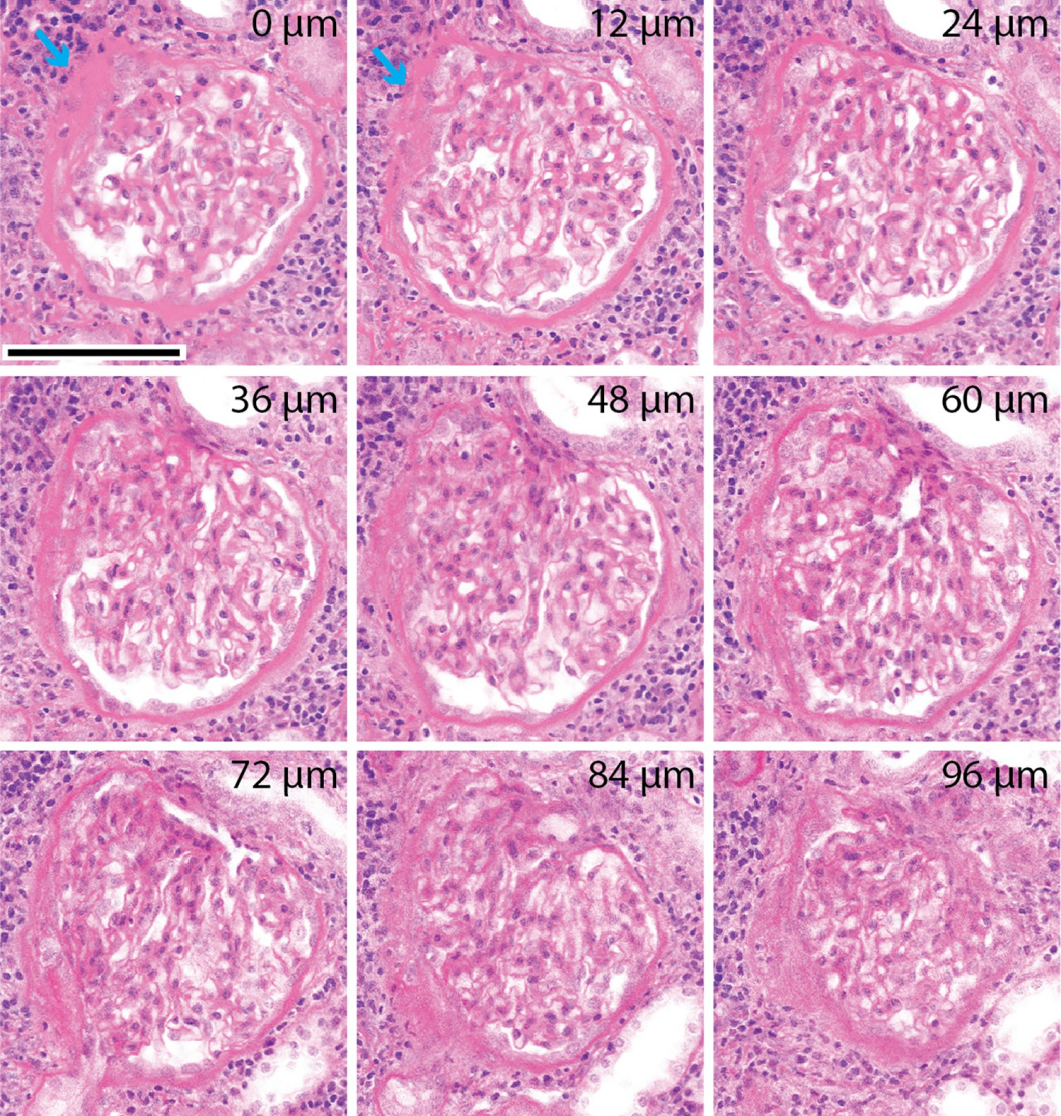
Focal and segmental glomerulosclerosis. NLM images of a patient with focal and segmental glomerulosclerosis were acquired every 4 μm in depth. Images spaced 12 μm apart in depth show a segmental obliteration of the glomerular tuft with sclerosis (blue arrow), adhesions and obliteration of the urinary space. The relationship to the vascular pole is seen only in certain levels and an adhesion to the urinary pole is seen only in one image (at 72 μm depth). The depth below the tissue surface is indicated in microns (top right). Scale bar = 100 μm.

**Fig 4:**
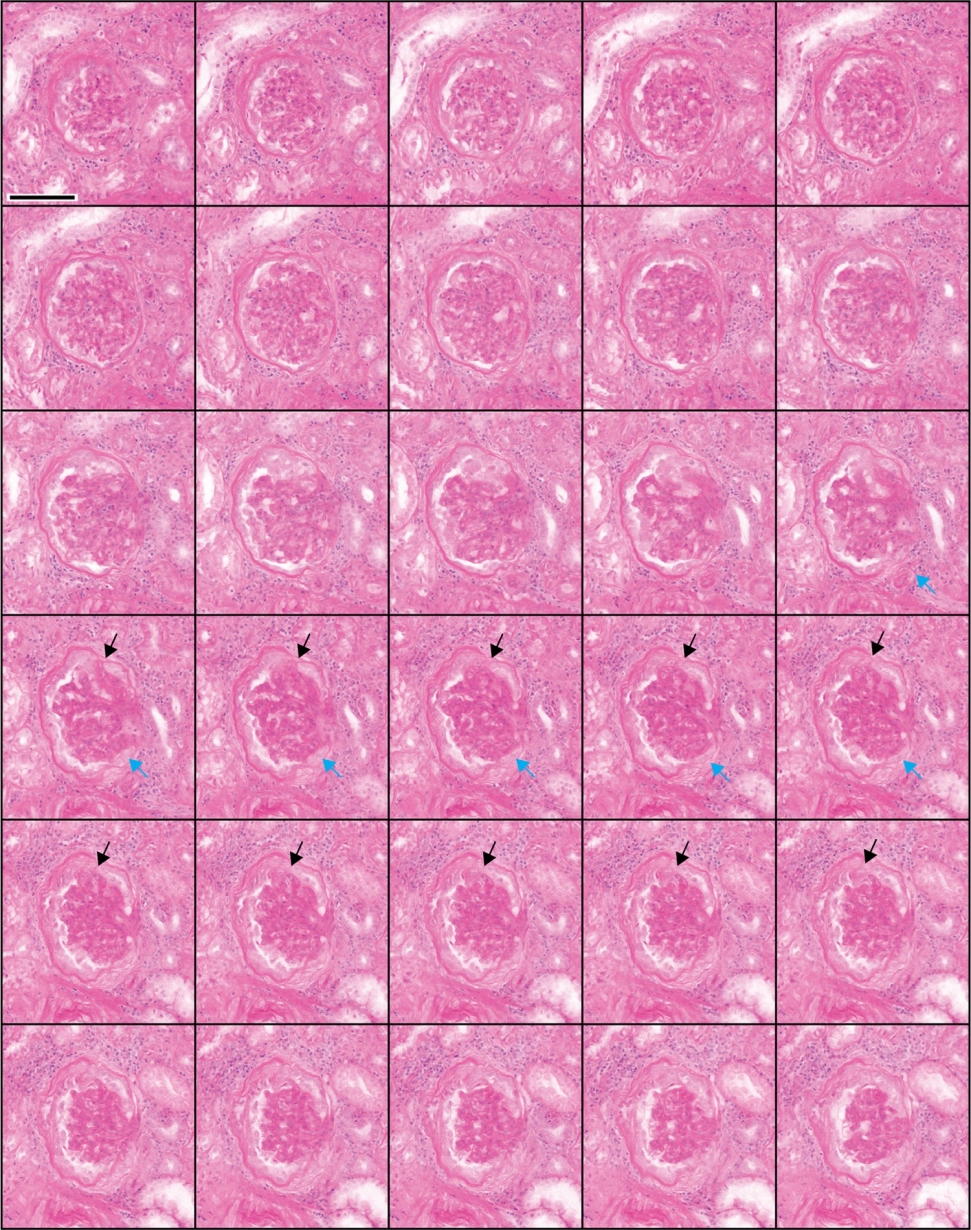
Three-dimensional NLM analysis of FSGS with features of collapsing glomerulopathy. NLM images spaced 4 μm in depth are shown (from left to right). Sections of glomerular collapse with associated epithelial proliferation and formation of synechia (black arrows) are seen. Fragmentation of Bowman’s capsule at the vascular pole is seen (blue arrows) associated with epithelial proliferation. Scale bar = 100 μm.

NLM with optical clearing enabled 3D evaluation of focal lesions such as in biopsies with FSGS. NLM images selected at 12 μm intervals throughout a 3D volume of a glomerulus are shown in Figs 3 and 4. At the first level (0 μm) in Fig 3, the glomerular tuft is largely maintained but the left upper aspect is sclerotic (blue arrow). At deeper levels, it becomes more obvious that the tuft is adherent to Bowman’s capsule which is accompanied by proliferation of Bowman’s capsular cells and synechiae. At deeper levels, the vascular pole and macula densa become well delineated. The mesangial proliferation and matrix increase appear more evident in the area of the tuft with the segmental changes. The accompanying chronic inflammatory infiltrate is seen at all levels.

#### IgA nephropathy

**Fig 5:**
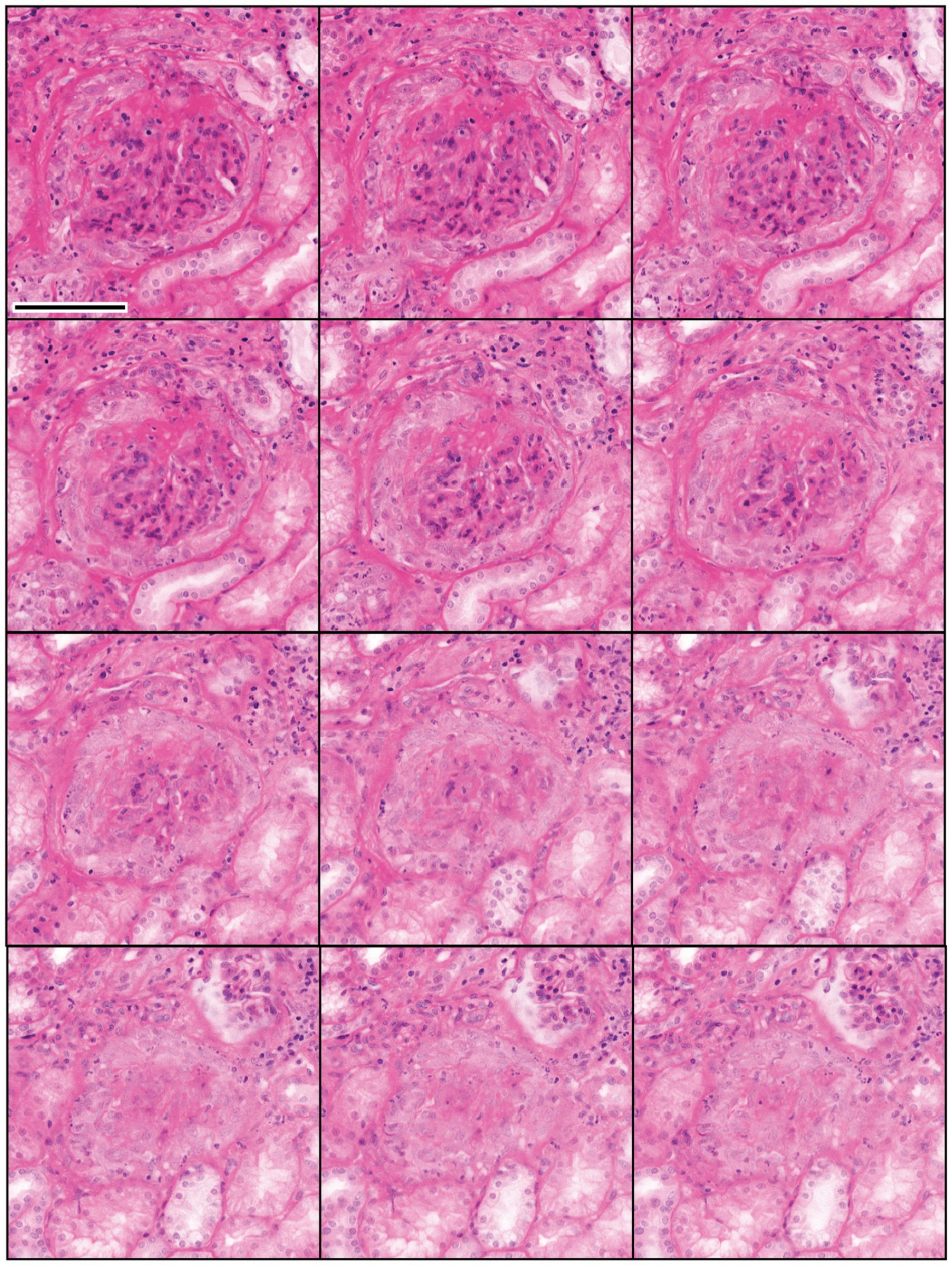
Three-dimensional NLM analysis of crescentic IgA nephropathy. NLM images spaced 4 μm in depth are shown (from left to right). In the first few images (upper left) the crescent extends into the proximal tubule. The crescent is global and associated with scattered neutrophils. Scale bar = 100 μm.

A biopsy from a patient with IgA nephropathy with crescentic lesions is shown in Fig 5. The crescent is seen in the NLM image extending into the proximal tubule. The crescent is global and associated with scattered neutrophils.

#### Acute tubular necrosis (acute kidney injury)

**Fig 6:**
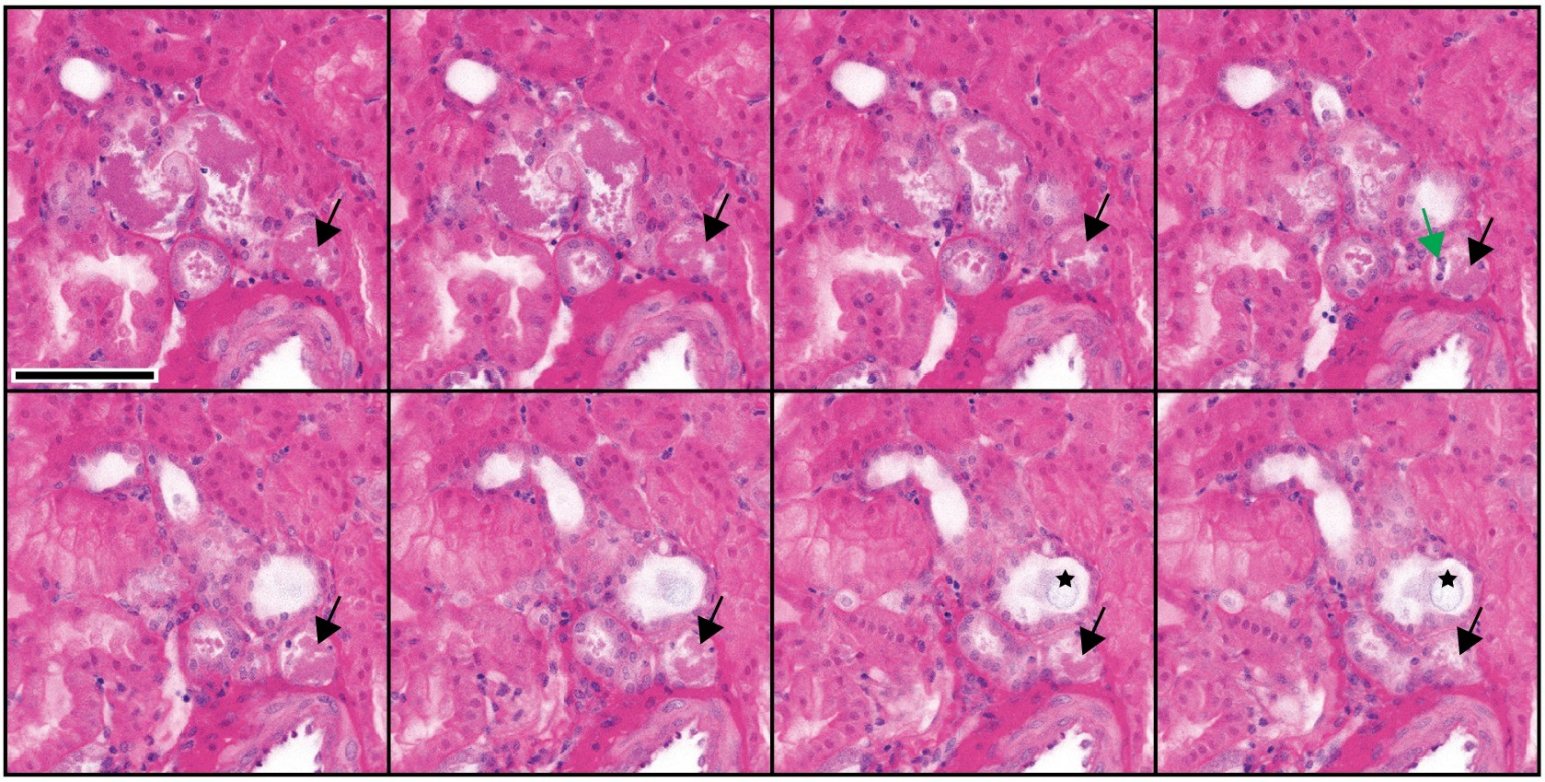
Three-dimensional NLM analysis of tubular necrosis–part 1. NLM images spaced 4 μm in depth are shown (from left to right). Proximal tubules appear intact. Distal tubules with granular debris, oxalate (star), necrosis (black arrow), and neutrophils (green arrow) are seen. Scale bar = 100 μm.

**Fig 7:**
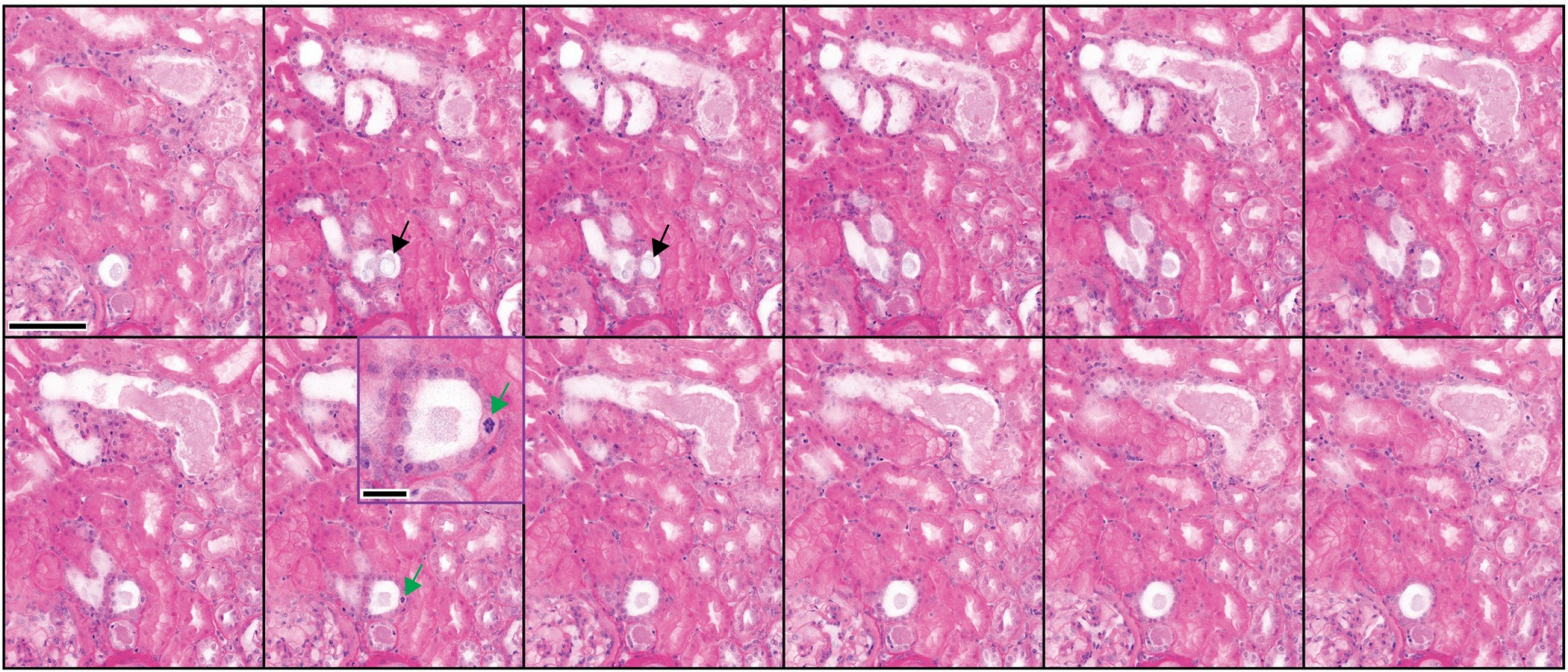
Three-dimensional NLM analysis of tubular necrosis–part 2. NLM images spaced 4 μm in depth are shown (from left to right). Proximal tubules appear intact. Deeper cortex with collecting ducts filled with granular debris, in which cellular elements can be identified. Oxalate is seen in a distal tubule (black arrow). A mitotic figure can be identified (green arrow). Scale bar = 100 μm. Inset scale bar = 25 μm.

Acute tubular necrosis (ATN) can be visualized with NLM. Figs 6 and 7 show granular debris in distal tubules and collecting ducts visualized with NLM. Neutrophils, necrosis, and oxalate are also apparent in 3D NLM images. Mitotic figures can be identified.

### Quantitation

#### Glomeruli

**Fig 8:**
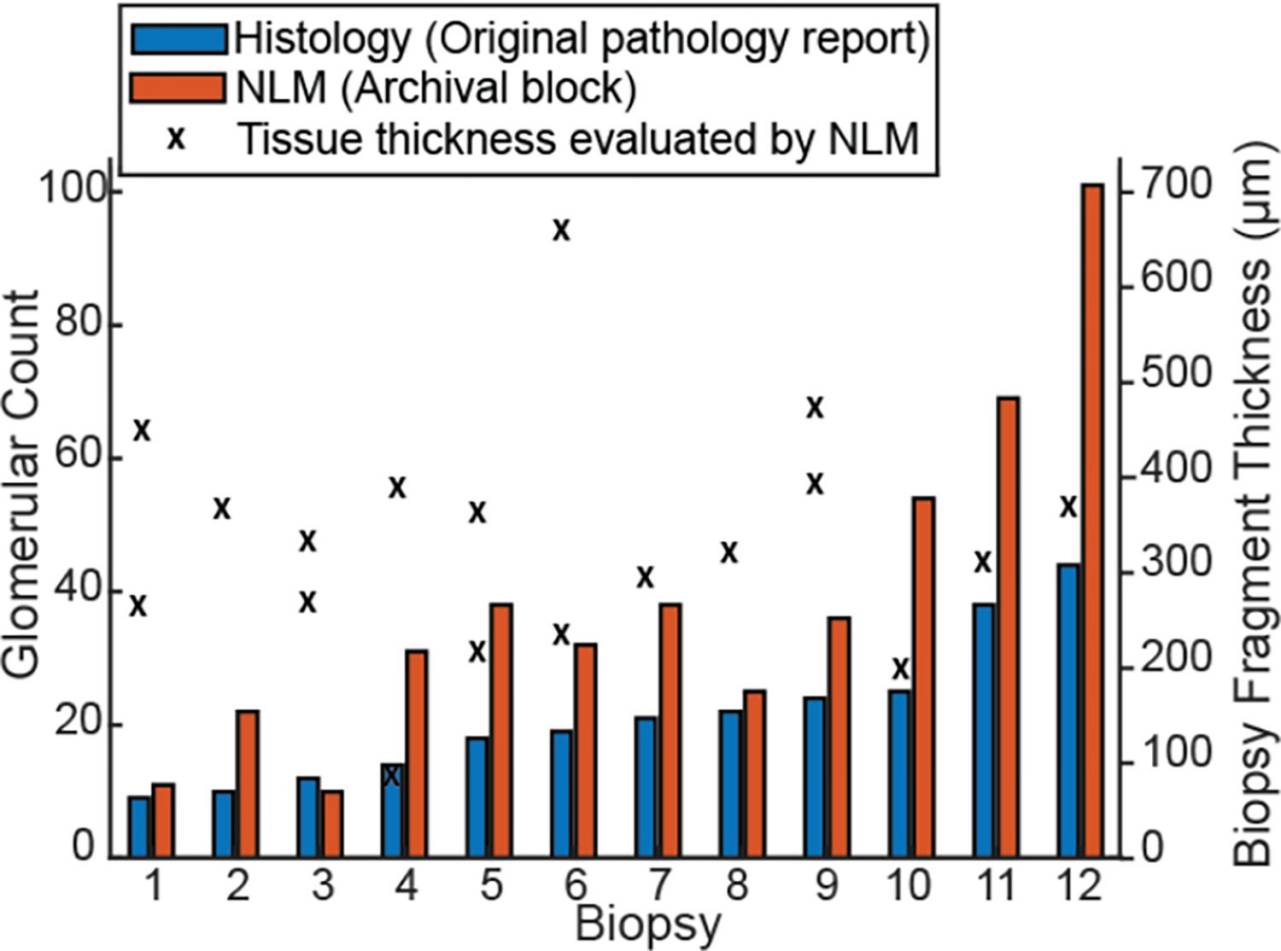
A plot of the glomerular count in the tissue analyzed using 3D NLM imaging versus the count on the original paraffin slides. The biopsies are ordered from least to greatest glomerular count. Note that the histology and NLM counts were performed on different tissue from the same biopsy. The count on histology was performed on the original histology slides while the count on NLM was performed on the tissue from the archival block that was remaining after cutting the histology slides. The biopsy tissue thickness evaluated by NLM is also shown. Several biopsies had multiple fragments in the paraffin block and therefore multiple thicknesses are shown.

Fig 8 compares the glomerular count from the 3D NLM volumes to the original paraffin histology. Note that the histology count was performed on the original histology slides while the NLM count was performed on tissue from the archival block. Furthermore, paraffin sections typically underestimate the glomerular count in renal biopsies [25]. The high glomerular count in the residual tissue indicates that archival blocks could be an important resource for retrospective studies.

**Fig 9:**
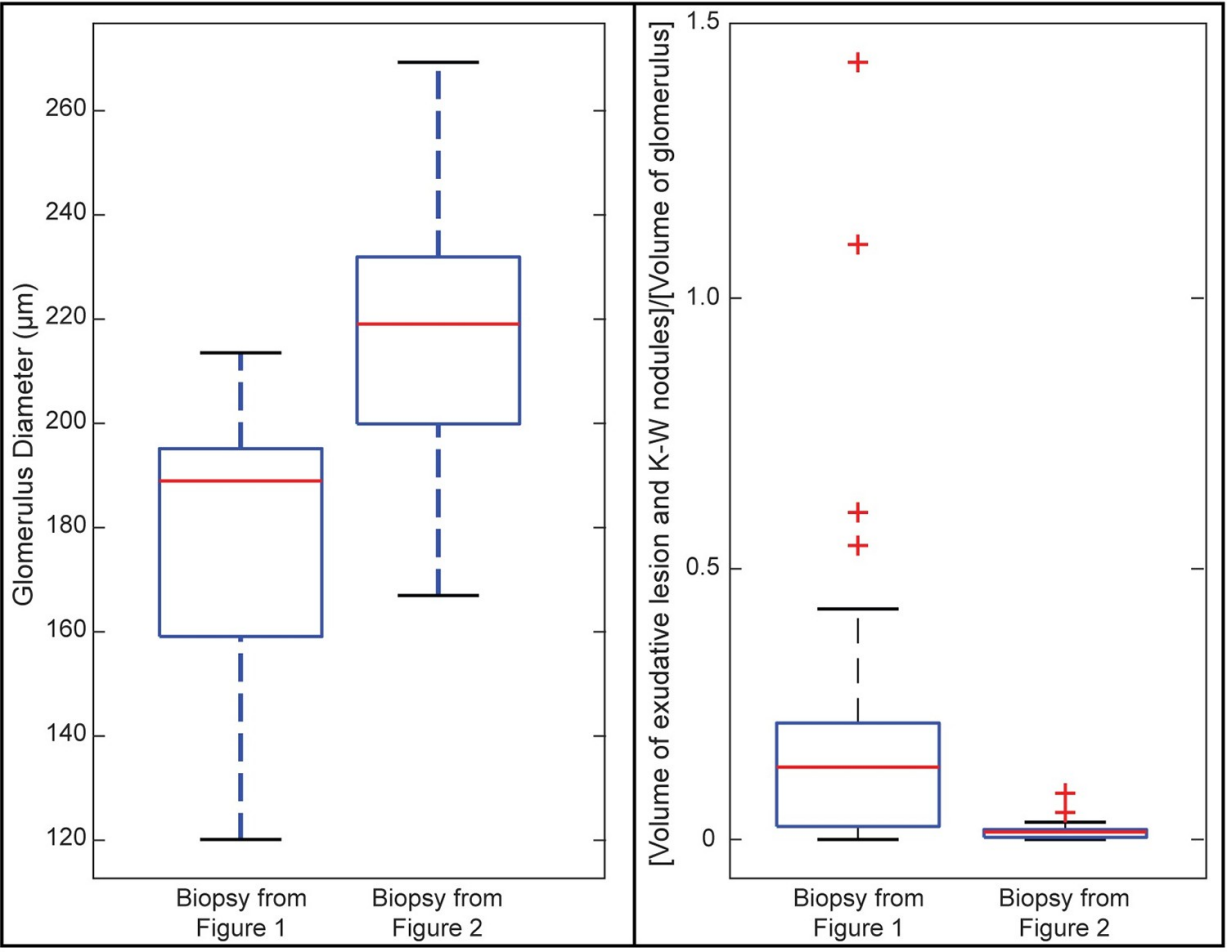
Box plot of glomerular diameters and lesional volumes in biopsies of patients with diabetic nephropathy. (**A**) A box plot of the diameters of all glomeruli from the biopsies shown in Figs 1 and 2. The mean glomerular diameters are 178.0 μm and 215.4 μm for the biopsy from Figs 1 and 2, respectively. (**B**) The volume of exudative lesions and Kimmelstiel-Wilson nodules in each glomerulus as a fraction of the volume the glomerulus is shown. The mean fraction of lesion volume:glomerular volume was 0.229 and 0.016 in the biopsy from Figs 1 and 2, respectively.

NLM analysis also enabled quantification that is difficult to perform with paraffin slides. For example, the biopsies in Figs 1 and 2, which were from patients with diabetic nephropathy, had mean glomerular diameters of 178.0 μm and 215.4 μm, respectively. The mean fraction of lesion volume (exudative lesions and Kimmelstiel-Wilson nodules) to glomerular volume was 0.229 in the biopsy in Fig 1 and 0.016 in the biopsy in Fig 2 (see Fig 9).

#### Tubulointerstitium

**Fig 10:**
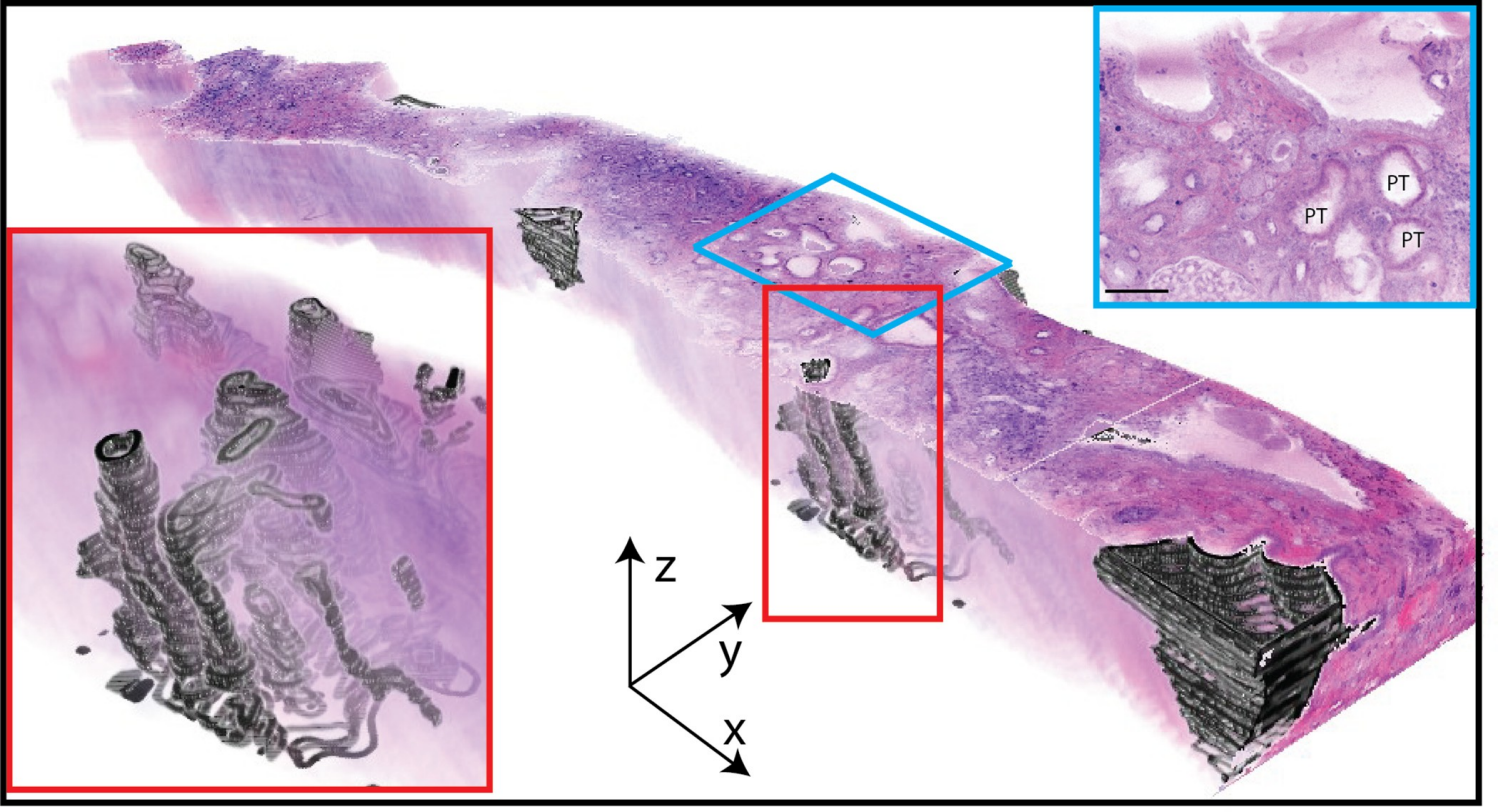
Three-dimensional NLM analysis of a biopsy from a patient diagnosed with lithium nephropathy. NLM images acquired every 4 μm in depth through the biopsy and reconstructed into a 3D volume. Individual cysts and tubules have been segmented in black and constitute 9.7% of the total biopsy volume. The 3D volume has been made partially transparent to visualize the segmented structures. A higher power view of the segmented tubules is shown in the red box. Hypertrophic proximal tubules (PT) are seen in the center and at higher power (blue box, 80 μm below biopsy surface, scale bar = 100 μm). Scale bar x, y, z = 100 μm.

Fig 10 shows a 3D NLM volume of a renal biopsy from a patient with lithium nephropathy. The extent of parenchymal compromise is seen best in 3D. The cysts were segmented and the hypertrophic proximal tubules are seen as convoluted structures (blue and red box). Cysts could also be quantified and were measured to be 9.7% of total biopsy volume. These measurements as well as spatial relationships within the glomeruli and biopsies could be readily obtained from 3D NLM volumes.

### Reversible processing

**Fig 11:**
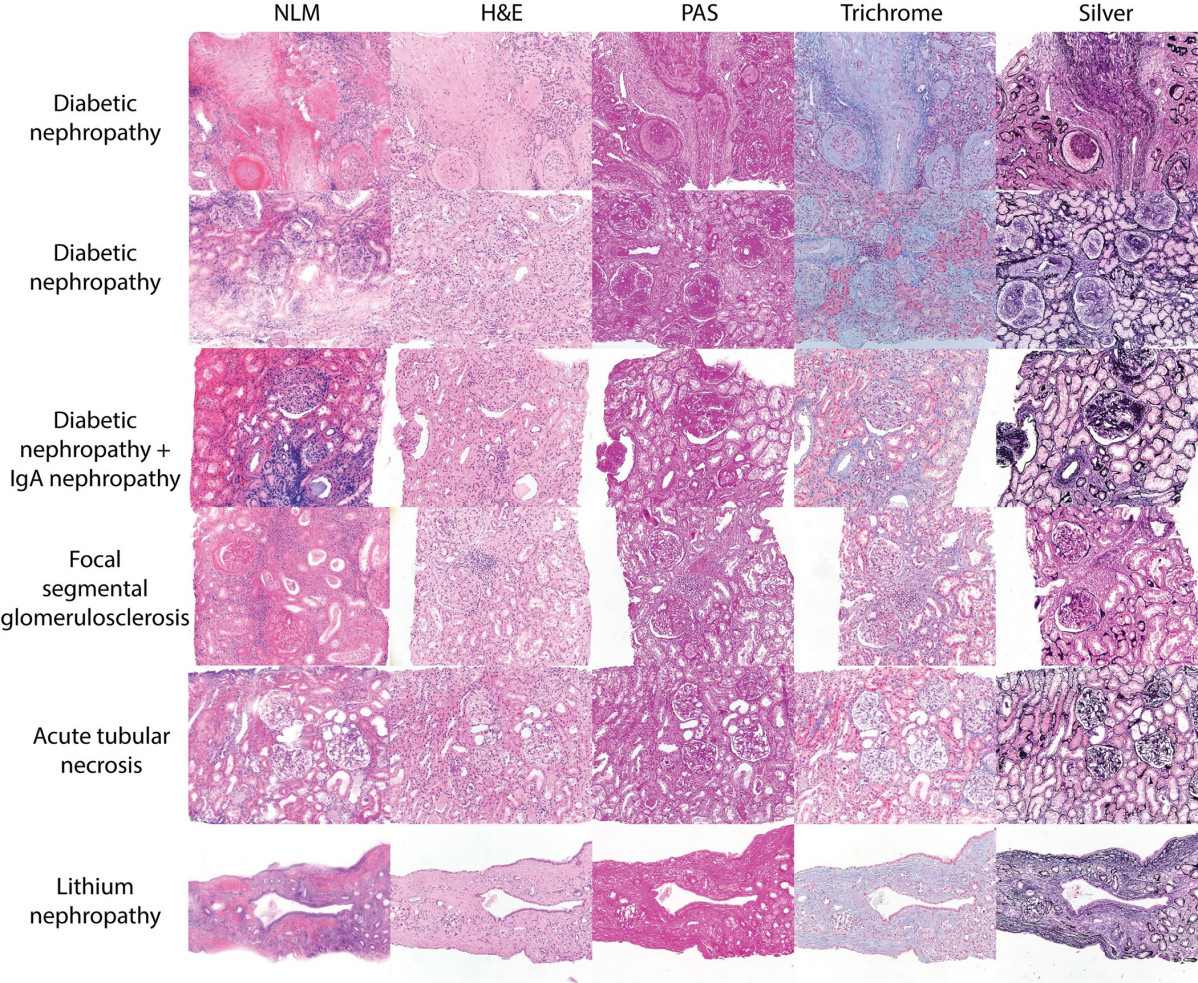
Histology of biopsies that were first optically cleared and evaluated with NLM. The first column shows NLM images from 6 different biopsies that were optically cleared. After tissue optical clearing and NLM imaging, biopsies were declarified, re-embedded in paraffin, and processed for standard evaluation. H&E, PAS, Masson’s trichrome, and Jones methenamine silver staining is not affected by prior NLM analysis.

After tissue optical clearing and NLM imaging, biopsies could be declarified, re-embedded in paraffin, and processed for standard evaluation. NLM imaging with optical clearing did not alter standard tissue processing (Fig 11).

## Discussion

In this study, we demonstrate an optical clearing, staining and 3D NLM imaging protocol for analysis of archival renal biopsies without microtome sectioning. The NLM images closely resemble paraffin H&E slides. Hypercellularity, mesangial expansion, hyalinized arterioles, and Kimmelsteil-Wilson nodules were clearly visualized in diabetic nephropathy. NLM enabled examination of segmental sclerosis, glomerular tuft adhesions, and mesangial proliferation in FSGS. Crescentic IgA nephropathy, oxalate, necrosis, and tubular granular debris in ATN, and large cysts in lithium nephrotoxicity are also apparent in NLM images.

There are differences between NLM images and paraffin H&E slides including decreased cell border sharpness, thicker appearing images, and variations in cytoplasmic color. In this initial study, the NLM images were acquired on thick tissue using a 20X, 1.0NA objective with a ~4 μm axial (z) resolution. Paraffin histology is typically cut at 2 μm and therefore NLM images appear thicker. Higher NA/power objectives can be used to provide higher resolution images at the cost of decreased imaging speed. Variations in cytoplasmic color likely result from differences in staining characteristics of Hoechst and Eosin versus Hematoxylin and Eosin. These differences are consistent across biopsies and patients and pathologists with experience and training may become comfortable interpreting these differences. Furthermore, standard paraffin evaluation can be performed in addition to NLM analysis.

In this paper, we show that 3D NLM evaluation enables visualization and quantification of disease markers including sclerotic lesions, Kimmelstiel-Wilson nodules, mitotic figures, necrosis, glomerular collapse, and cystic changes. It is important to note that NLM evaluation is not meant to replace current standard-of-care tissue studies (Periodic acid-Schiff (PAS), Jones methenamine silver, Masson’s Trichrome, immunofluorescence) but rather supplement them. NLM evaluation enables more precise analysis of known diagnostic, therapeutic, and prognostic indicators. Larger studies will be required to investigate if NLM could provide additional insight into disease diagnosis, progression or treatment response.

Evaluating biopsies in 3D could be advantageous in many focal diseases. Differentiating between minimal change disease and FSGS is important for predicting therapeutic response and disease prognosis [2]. The more glomeruli that are evaluated in paraffin histology, the higher possibility that a glomerulus affected by FSGS will be found [4,5]. ANCA glomerulonephritis (GN), lupus nephritis, and diabetic nephropathy also often present with focal components of injury [6,10].

There are several limitations to this study. This study has a small sample size and the biopsies analyzed represent a limited number of diseases. Larger studies will be required for further characterization of this technique. Furthermore, a limited number of known diagnostic and prognostic indicators were analyzed. The staining and imaging technique demonstrated in this paper is limited to a nuclear and cytoplasmic/stromal fluorescent stain analogous to hematoxylin and eosin. Techniques that simulate stains such as PAS, Jones methenamine silver, and Masson’s trichrome may improve the technique. However, as presented, this technique in not meant to replace standard tissue studies but augment them. In addition, the NLM instrument used in this study is a prototype currently being developed for wider clinical use.

Previous studies have demonstrated analysis of needle biopsies from autopsy kidneys using NLM with similar clearing techniques [21]. The studies presented in this paper extend these findings by demonstrating that archival tissue can be analyzed by optically clearing and NLM imaging, showing common markers of renal disease on deparaffinized archival renal biopsies, and illustrating that archival biopsies present a rich source of previously unanalyzed tissue. Large stores of archival paraffin blocks are available with comprehensive clinical data on outcomes. Retrospective analysis of these specimens will be required to further assess the utility of NLM in biopsy analysis.

## Supporting information

S1 Data (DOCX)
