# Supplementary material for: Data retrieval from archival renal biopsies using nonlinear microscopy
Source: PLoS One. 2024 Mar 15;19(3):e0299506. doi: 10.1371/journal.pone.0299506 (PMC10942027; doi:10.1371/journal.pone.0299506)
Supplement: S1 Data — (DOCX) [file pone.0299506.s001.docx]

**Data for Figure 8**

| Biopsy # | Tissue thickness evaluated by NLM (µm) | Histology Glomerular Count | NLM Glomerular Count |
| --- | --- | --- | --- |
| 1 | 268 and 448 | 9 | 11 |
| 2 | 368 | 10 | 22 |
| 3 | 264 and 332 | 12 | 10 |
| 4 | 88 and 388 | 14 | 31 |
| 5 | 216 and 380 | 18 | 38 |
| 6 | 232 and 660 | 19 | 32 |
| 7 | 292 | 21 | 38 |
| 8 | 320 | 22 | 25 |
| 9 | 392 and 472 | 24 | 36 |
| 10 | 200 | 25 | 54 |
| 11 | 312 | 38 | 69 |
| 12 | 368 | 44 | 101 |

**3D Data for figures within the main text**

Data has been deposited in Dryad (https://doi.org/10.5061/dryad.pzgmsbctz).
